# Variation in Lipid Components from 15 Species of Tropical and Temperate Seaweeds

**DOI:** 10.3390/md17110630

**Published:** 2019-11-06

**Authors:** Eko Susanto, A. Suhaeli Fahmi, Masashi Hosokawa, Kazuo Miyashita

**Affiliations:** 1 Laboratory of Bio-resources Chemistry, Graduate School of Fisheries Sciences, Hokkaido University, Hakodate 041-8611, Japan; hoso@fish.hokudai.ac.jp (M.H.); kmiya@fish.hokudai.ac.jp (K.M.); 2 Department of Fisheries Products Technology, Faculty of Fisheries and Marine Science, Diponegoro University, Jl. Prof. Soedarto SH Kampus Tembalang, Semarang 50275, Republic of Indonesia; suhaeli.fahmi@live.undip.ac.id

**Keywords:** seaweeds, chlorophylls, carotenoids, *n-3* PUFAs, EPA

## Abstract

The present study describes the variation in lipid components from 15 species of seaweeds belonging to the Chlorophyta, Ochrophyta, and Rhodophyta phyla collected in tropical (Indonesia) and temperate (Japan) areas. Analyses were performed of multiple components, including chlorophylls, carotenoids, *n-3* and *n-6* polyunsaturated fatty acids (PUFAs), and alpha tocopherol (α-Toc). Chlorophyll (Chl) and carotenoid contents varied among phyla, but not with the sampling location. Chl a and b were the major chlorophylls in Chlorophyta. Chl a and Chl c were the main chlorophylls in Ochrophyta, while Chl a was the dominant chlorophylls in Rhodophyta. β-Carotene and fucoxanthin were detected as major seaweed carotenoids. The former was present in all species in a variety of ranges, while the latter was mainly found in Ochrophyta and in small quantities in Rhodophyta, but not in Chlorophyta. The total lipids (TL) content and fatty acids composition were strongly affected by sampling location. The TL and *n-3* PUFAs levels tended to be higher in temperate seaweeds compared with those in tropical seaweeds. The major *n-3* PUFAs in different phyla, namely, eicosapentaenoic acid (EPA) and stearidonic acid (SDA) in Ochrophyta, α-linolenic acid (ALA) and SDA in Chlorophyta, and EPA in Rhodophyta, accumulated in temperate seaweeds. Chlorophylls, their derivatives, and carotenoids are known to have health benefits, such as antioxidant activities, while *n-3* PUFAs are known to be essential nutrients that positively influence human nutrition and health. Therefore, seaweed lipids could be used as a source of ingredients with health benefits for functional foods and nutraceuticals.

## 1. Introduction

The consumption of seaweeds has existed for millennia, not only in Japan, Korea, and China, but also in some Southeastern Asian countries, such as Malaysia, the Philippines, and Indonesia [1]. Recently, global seaweed production has risen considerably with more than 291 exploited species for food, feed, paper, fertilizer, medicinal, and industrial product uses [2]. This might be because of the increasing interest in seaweeds as nutraceuticals, functional foods, cosmetics, and pharmaceuticals because of the presence of characteristic nutrients and bioactives in seaweeds [3].

Seaweeds, promising marine products owing to their sustainability, contain valuable bioactive compounds that possess potential benefits for human health, such as anti-obesity, anti-diabetes, anticancer, and cardioprotective activities [4,5,6,7,8,9,10,11,12,13]. The most abundant nutrients in seaweeds are non-starch polysaccharides and minerals. The lipid content of seaweeds is low (1%–5% of the weight of a dry weight (DW) sample); however, the lipids comprise many kinds of bioactives, such as *n-3* and *n-6* polyunsaturated fatty acids (PUFAs), chlorophylls, carotenoids, terpenoids, and sterols [14,15,16,17,18].

Seaweeds are marine algae that are rich sources of lipid soluble pigments, such as chlorophylls and carotenoids. These natural pigments from seaweeds show several biological activities. Specifically, there have been many studies on the nutritional properties of seaweeds’ carotenoids, such as fucoxanthin (Fx) [19]. Fx is a specific carotenoid found in brown seaweeds and known to help manage obesity and type 2 diabetes mellitus [20]. Chlorophylls and their various derivatives have a long-established history of use in traditional medicine and for therapeutic purposes [21]. Antioxidant and antitumor activities have been reported as general physiological activities of chlorophylls and their derivatives [21,22]. In addition, chlorophylls from seaweeds exhibit therapeutic effects, such as anti-degranulation properties in RBL-2H3 cells [23,24], anti-inflammatory activity in RAW 264.7 cells [25,26], and neuroprotective activity in PC12 cells [27]. Because of their valuable health benefits, these natural pigments have attracted interest in their potential application in functional foods, cosmetics, and pharmaceuticals [28], while more efforts are needed to clarify the detailed biological activities of chlorophylls from seaweeds. 

In addition to the pigments, seaweed lipids generally contain high levels of PUFAs, such as α-linolenic acid (18:3*n-3*, ALA), stearidonic acid (18:4*n-3*, SDA), and eicosapentaenoic acid (20:5*n-3*, EPA) as *n-3* PUFAs, and arachidonic acid (20:4*n-6*, ARA) as an *n-6* PUFAs [14,16,18,29]. Many studies, including substantial epidemiological, case-control, clinical, genetic, and nutrigenetic approaches, demonstrate a reduction in cardiovascular disease risk from intake of *n-3* PUFAs, such as EPA and docosahexaenoic acid (22:6*n-3*, DHA), which are the active forms of *n-3* PUFAs [16,30]. ARA also plays an important role in biological systems, such as in the immune response, thrombosis, and brain function [16]. ARA and DHA are used as supplements in commercial infant formulas because both PUFAs are essential for infant neurodevelopment. Furthermore, the combination of ARA and DHA has also been found to improve age-related disorders of the brain and cognitive function [31]. 

Tropical seaweed species have significantly lower lipid content than cold-water species [16]. Comparative studies have revealed that the total lipids (TL) content of a major brown seaweed family, Sargassaceae, was higher in subarctic zones (approximately 5% of the DW) than in tropical zones (0.9%–1.8% of the DW) [14,32,33,34]. In addition, different seaweed species have different TL contents, and some species have shown exceptionally high TL contents. For example, Gosch et al. [35] found higher TL contents in three tropical brown seaweed species (10.80%–11.91% of the DW) and two tropical green seaweed species (12.14%–13.04% of the DW) collected in tropical North Queensland, Australia. Higher TL contents have also been reported in brown seaweeds collected in the Indian Ocean (7%–8% of the DW) [36], as well as the Hawaiian coast (16%–20% of the DW) [37]. Therefore, seaweed lipids content varies greatly by species, geographical location, season, temperature, salinity, and light intensity, as well as interactions among these factors [14,34,38,39,40,41,42]. The content of lipid-soluble bioactive compounds in seaweeds also depends on several factors, such as species, sampling sites, environment, and seasonal variation [14,34,37,38,39,40,41,42,43,44,45,46,47]. 

Although many papers have reported variations in the lipid components from different species of seaweeds collected in various areas, most of them have focused on the fatty acids composition [48,49,50,51] and on specific bioactives, such as Fx [14,34,38]. In finding a potential target in seaweed as an ingredient for foods, animal feed, cosmetics, and nutraceuticals, information on the wide range of lipid and related compound profiles, especially on lipid soluble pigments, will be required. Among seaweed pigments, chlorophylls play a key role in photosynthesis, while carotenoids serve as antenna pigments by absorbing and passing light energy to chlorophyll. Both pigments are not only essential for photosynthesis, but also show various health benefits, as described above. However, little information is available on both lipid soluble pigment levels in seaweeds. The investigation of lipid-soluble compounds in seaweed grown near the coast of Indonesia and northern coast of Japan is limited [34].

Therefore, the primary aim of this study was to determine chlorophylls and carotenoids in 15 different species of seaweeds belonging to Ochrophyta (brown seaweeds), Chlorophyta (green seaweeds), and Rhodophyta (red seaweeds). In addition, the present study also revealed the fatty acids and α-tocopherol (α-Toc) profiles of these different seaweeds. Each sample was collected from tropical (Indonesia) or temperate (Japan) waters. The present study will be useful for the comparison between the characteristics of lipid soluble compounds from seaweeds collected from the two different areas. 

## 2. Results

### 2.1. Total Lipids

The TL of seaweeds from Indonesia (tropical area) and Japan (temperate area) are described in Table 1. The TL level varied among species, ranging from 6.91 to 62.48 mg∙g^−1^ DW. On average, the highest TL content was recorded in Ochrophyta (42.44 mg∙g^−1^ DW), followed by Chlorophyta (34.60 mg∙g^−1^ DW), and Rhodophyta (17.75 mg∙g^−1^ DW). In the present study, the lowest TL content was reported in *Chondrus yendoi* (6.91 mg∙g^−1^ DW), while *Ulva australis* had the highest total lipids content among all seaweeds tested.

### 2.2. Pigments

The primary photosynthetic pigments in seaweed are comprised of chlorophylls and carotenoids. In this study, we investigated the variation of these pigment contents in different phyla of seaweeds, which were collected from two regions (Table 2 and Table 3). Chlorophylls easily decompose to produce their derivatives, pheophytins, by elimination of magnesium. This change from chlorophylls to pheophytins is accelerated by heat treatment. In the dried samples from tropical seaweeds, chlorophyll a (Chl a) and chlorophyll b (Chl b) levels were very low or undetected, while their corresponding derivatives, pheophytin a (Phy a) and pheophytin b (Phy b), were major chlorophyll components in these seaweeds. The lower level of Chl a and Chl b in the tropical seaweeds was derived from the decomposition of these chlorophylls to corresponding pheophytin derivatives during the drying process. 

Although Chl a was detected in all seaweeds from the temperate area, its level widely ranged from 1.74 (*Chondria crassicaulis*) to 268.82 (*Sargassum horneri*) mg∙100 g^−1^ DW. Chlorophyll b, the characteristic pigment in Chlorophyta, was present in small amounts and ranged from 16.83 (*Caulerpa lentillifera*) to 101.50 (*U. intestinalis*) mg∙100 g^−1^ DW. A unique finding was the occurrence of chlorophyll c (Chl c), the characteristic accessory pigment in Ochrophyta. It was not only found in Ochrophyta, but also in Rhodophyta (*C. crassicaulis* and *Mazaella japonica)*. Phy a was abundantly found in seaweeds of the Laminariaceae family and Rhodomelaceae family, while Phy b was only found in Chlorophyta. 

Carotenoids, accessory pigments in photosynthesis, also varied among seaweed species (Table 3). Provitamin A carotenoids, α-carotene (α-Car) and β-carotene (β-Car), exhibited different patterns. β-Car was present in all species with a range from 1.12 to 58.24 mg∙100 g^−1^ DW, while α-Car was detected only in Chlorophyta and Rhodophyta. Fx was mainly found in Ochrophyta and found in a small amount in Rhodophyta, but not in Chlorophyta. In addition, Zeaxanthin (Zx) content was relatively higher in two *Sargassum* species (*S. horneri* and *S. fusiforme*) and *Undaria pinnatifida* collected from the temperate area. Lutein (Lut) was found in Rhodophyta and Chlorophyta with a range from 0.62 mg∙100 g^−1^ DW (*M. japonica*) to 124.82 mg∙100 g^−1^ DW (*U. australis*), but was not found in Ochrophyta. Violaxanthin (Vx) was only found in Ochrophyta and Chlorophyta collected from Japan with a range from 1.45 mg∙100 g^−1^ DW (*Costaria costata*) to 23.30 mg∙100 g^−1^ DW (*U. australis*). Neoxanthin, only present in Chlorophyta, also showed a wide range in its content from 30.63 mg∙100 g^−1^ DW (*U. reticulata*) to 185.56 mg∙100 g^−1^ DW (*U. australis*). Higher total carotenoid levels were found in Ochrophyta (*S. aquifolium*, *C. costata*, *S. japonica*, *U. pinnatifida*, *S. fusiforme*, *S. horneri*) from the two different areas. Chlorophyta species collected from the temperate area (*U. australis* and *U. intestinalis*) also showed higher total carotenoid levels, whereas Chlorophyta from the tropical area (*C. lentillifera* and *U. reticulata*) showed relatively low levels. The same trend was also found among Rhodophyta species collected from the temperate area (*C. crassicaulis*, *C. yendoi*, *Gloiopeltis furcata*, *M. japonica*) and those collected from the tropical area (*Gracilariopsis longissima*).

### 2.3. Fatty Acids

The details of fatty acids composition in seaweed lipids are listed in Table 4 and Tables S1a, S1b and S1c. All values are expressed as percentages of total fatty acids (FAs) content. The absolute content of total *n-3* PUFAs (mg∙g^−1^ DW) is also shown in Table 4. The fatty acids composition varied widely among seaweed species and sampling locations.

In the three phyla, Ochrophyta, Chlorophyta, and Rhodophyta, palmitic acid (C16:0) was the predominant fatty acids (Tables S1a, S1b and S1c). The temperate seaweeds contained much more total PUFAs (28.15% (*C. crassicaulis*) to 51.28% (*S. japonica*)) than the tropical seaweeds (13.06% (*C. lentillifera*) to 25.75% (*U. reticulata*)) (Table 4). Specifically, the absolute amounts of total *n-3* PUFAs in the temperate seaweeds were much higher than those in the tropical seaweeds. Total saturated fatty acids (SFAs) contents in the tropical seaweeds were relatively higher than those in the temperate seaweeds, except for *M. japonica*. Furthermore, monounsaturated fatty acids (MUFAs), dominantly comprising C16:1*n*-7 and C18:1*n*-9, were higher in temperate Rhodophyta, namely, *C. crassicaulis*. Overall, in the tropical seaweeds, SFAs accounted for 29.80% to 47.78%, followed by PUFAs (13.06% to 25.75%) and MUFAs (6.88% to 18.88%), while PUFAs (36.23% to 51.28%) were the most dominant FA class in the temperate seaweeds, except for *C. crassicaulis* (28.15%) and *M. japonica* (31.21%). Furthermore, Chlorophyta contained relatively less C20 FAs; however, C20 FAs were the major FAs in Ochrophyta (*S. horneri, S. fusiforme*) and in Rhodophyta, especially those collected in the temperate area (Table 4). 

Total PUFAs levels varied among species, ranging from 13.06% (*C. lentillifera*) to 51.28% (*S. japonica*) of total fatty acids. These seaweeds contained EPA (C20:5*n-3*), ARA (C20:4*n-6*), ALA (C18:3*n-3*), and SDA (C18:4*n-3*) as the major PUFAs; however, there was significant variation in the level of these PUFAs in the TL content from each species. The main PUFAs in Ochrophyta collected from the temperate area were ARA (10.55% (*U. pinnatifida*) to 14.87% (*S. horneri*)) and EPA (8.36% (*C. costata*) to 13.04% (*S. japonica*)), whereas the main PUFAs in Ochrophyta collected from the tropical area (*S. aquifolium*) was ALA (10.40%), and the EPA level of this seaweed was very low (0.96%) (Table S1a). The same trend was found in Rhodophyta (Table S1c). Rhodophyta collected from the temperate area contained relatively high levels of EPA (13.08% (*C. crassicaulis*) to 35.81% (*G. furcata*)) and of ARA (3.33% (*C. crassicaulis*) to 17.26% (*C. yendoi*)); however, the main PUFAs of Rhodophyta collected in the tropical area (*G. longissima*) was ARA (11.41%), and the EPA level was 0.21%. Chlorophyta collected from the two geographical locations contained ALA as its major PUFAs (Table S1b). Among the 15 seaweeds analyzed, *G. furcata* exhibited the highest value of total *n-3* PUFAs (38.11%) with 35.81% EPA, whereas *U. intestinalis* exhibited the highest value of *n-3* PUFAs expressed in mg∙g^−1^ DW (9.84 mg∙g^−1^ DW) (Table 4). 

### 2.4. Nutritional Quality Index

The nutritional quality index based on the fatty acids composition was calculated in this study (Table 5). The *n-6/n-3* ratio was less than 3, except for *G. longissima* (7.69), and most of the ratios were less than 1.0. In general, the atherogenicity index (AI) and thrombogenicity index (TI) values increase with the decreasing degree of unsaturation of FAs, while the fatty acids hypocholesterolemic/hypercholesterolemic ratio (h/H) increases with the increasing unsaturated fatty acids composition. Therefore, AI and TI tended to be higher in the tropical seaweeds compared with those in temperate seaweeds. The h/H ratios of all temperate seaweeds were higher than those of the tropical seaweeds. In addition, the unsaturation index (UI) in all seaweeds was more than 3, and in Rhodophyta collected in the temperate area, it was more than 4.

### 2.5. Alpha Tocopherol

α-Toc content varied with seaweed species (Table 6). Relatively lower α-Toc levels were found in Chlorophyta (*C. lentillifera*, *U. reticulata*, *U. australis*, *U. intestinalis*). Seaweeds belonging to Ochrophyta and Rhodophyta showed relatively higher levels of α-Toc, except for *C. crassicaulis*. Specifically, *C. yendoi* showed the highest α-Toc content (9.34 mg∙100 g^−1^), followed by two species of *Sargassum* (*S. fusiforme* and *S. horneri*).

### 2.6. Multivariate Analysis 

**Figure 1:**
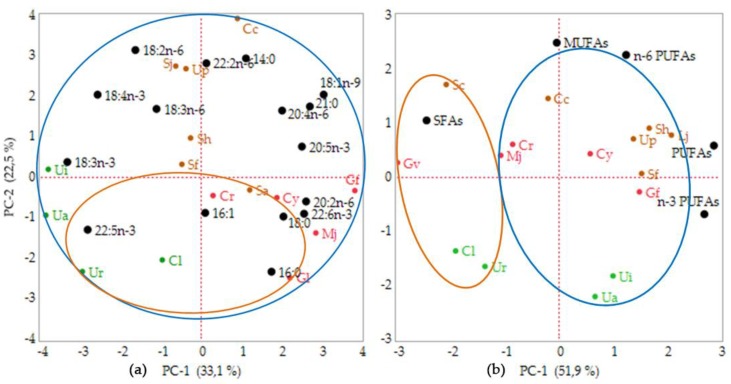
Bi-plot of the seaweeds’ sample received from PCA analysis of (**a**) fatty acids (FAs) data composition (15 fatty acids), (**b**) FAs group (SFAs, MUFAs, PUFAs, *n*-3 PUFAs, and *n*-6 PUFAs). Abbreviations: Cl: *Caulerpa lentillifera*; Ua: *Ulva australis*; Ui: *Ulva intestinalis*; Ur: *Ulva reticulata*; Cc: *Costaria costata*; Up: *Undaria pinnatifida*; Sj: *Saccharina japonica*; Sa: *Sargassum aquifolium*; Sf: *Sargassum fusiforme*; Sh: *Sargassum horneri*, Cr: *Chondria crassicaulis*; Cy: *Chondrus yendoi*; Gf: *Gloiopeltis furcata*; Gl: *Gracilariopsis longissima*; Mj: *Mazzaella japonica*. The seaweeds species codes are colored based on the seaweeds’ phyla (green: Chlorophyta; brown: Ochrophyta; red: Rhodophyta). The fatty acids composition and FAs group variable are colored in black. The circles were drawn based on geographical sampling location (orange circle: tropical seaweeds; blue circle: temperate seaweeds).

A statistical analysis was performed using the 15 fatty acids composition measured in this study in order to establish the relationship between geographical location and phylum. The fifteen FA variables explained the variability presented in the data, with principle component 1 (PC-1) accounting for 33.1% and principle component 2 (PC-2) for 22.5% of the variation (Figure 1a). Although the total percentage was only 55.6%, the analysis revealed a correlation of each seaweed within the phyla. Axis I separated the Rhodophyta species from the Chlorophyta, while Ochrophyta was positioned intermediately between these two, based on the principle component analysis (PCA) shown in the bi-plot data. However, these results could not determine whether the geographical location affected the fatty acids composition. Therefore, the PCA on the FA groups was analyzed. The bi-plot of the FA groups’ data matrix showed 81.5% of variance with PC-1 contributing 51.9% and PC-2 contributing 22.5% (Figure 1b). These data exhibited a broad diversity of fatty acids in the seaweed samples. The analysis revealed that the tropical seaweeds were rich in SFAs, while temperate seaweeds were dominated by PUFAs, including *n-3* and *n-6* PUFAs, excluding *C. costata*, *M. japonica*, and *C. crassicaulis*. When PCA was used for clustering of the fatty acids group, the seaweeds could be grouped within the sampling location. 

## 3. Discussion

Seaweeds have been an integral part of Japanese cuisine for the past one hundred years, similar to the diet of coastal communities in several Indonesian regions [52,53]. Industries use seaweeds as marine hydrocolloids and ingredients for foods and animal feed. Utilization of seaweeds as a dietary staple is because of their characteristic nutrients, which are shown to have therapeutic effects in humans [28,54,55,56,57]. Seaweeds are a source of novel bioactive compounds, such as certain polysaccharides and antioxidants, which are not found in terrestrial plants. The consumption of seaweeds has been linked to a lower incidence of chronic diseases, such as cancer, hyperlipidemia, and coronary heart disease [58]. Recently, seaweed lipids have drawn increased interest because of the presence of many kinds of bioactives, such as *n-3* and *n-6* PUFAs, carotenoids, and sterols [16]. Although seaweeds have a lower lipid content than marine fish, they are still a potential source of functional lipids because of their large stock in coastal waters.

Among bioactive seaweed lipids, much attention has been paid to the lipid soluble pigments, such as chlorophylls and carotenoids [28]. In addition, seaweed lipids are known to be rich in *n-3* and *n-6* PUFAs. However, little information has been available on the relationship between the multiple lipid bioactives from different species of seaweed. The present study analyzed the lipids from 15 different species of seaweed collected from two different regions, tropical (Indonesia) and temperate (Japan) regions, with an emphasis on the content of potentially bioactive components, chlorophylls, carotenoids, *n-3* and *n-6* PUFAs, and α-Toc. The results confirmed that the TL content and composition varied widely among species. Moreover, TL was sometimes affected by the location. As shown in Table 1, the TL level was higher in the temperate seaweeds than in the tropical seaweeds, although there were exceptions. It is generally accepted that the TL content of seaweeds varies with temperature, salinity, and light intensity. Specifically, the temperature strongly affects the TL level of seaweeds. Several studies have revealed that the TL content in temperate seaweeds is much higher than that in tropical seaweeds. The TL variation observed in the present study is consistent with previous studies [14,33,34,46].

Although the major components of the seaweed TL content are glycerolipids, such as glycoglycerolipids [16], other kinds of lipid-related compounds, such as photosynthetic pigments, are also abundant [28]. Chlorophylls and carotenoids are representative and major photosynthetic pigments found in seaweeds [59]. Chlorophylls are the most abundant pigments on earth and allow seaweeds to convert light into biological energy [22]. Carotenoids also have an important function as light energy harvesters in photosynthesis by passing on light excitation to chlorophylls. In addition, carotenoids act as antioxidants that inactivate reactive oxygen species formed by exposure to light and air during photosynthesis. Both lipid soluble pigments have been known to show a variety of nutritional effects in humans [20,25,26,28,60].

In seaweeds, the most important chlorophyll is Chl a, which absorbs the energy from the wavelength of violet blue and orange-red light [61]. Chl b and Chl c are also important chlorophylls found in seaweeds. They are accessory pigments in the antenna system of Chl a [62,63,64,65]. The present study revealed that the variation of these three kinds of chlorophylls was mainly affected by phylum, namely, Ochrophyta, Chlorophyta, and Rhodophyta, but not by sampling location (Table 2). In addition, the study also showed less Chl a and Chl b in the tropical seaweeds compared with those in the temperate seaweeds, while considerable amounts of the corresponding pheophytin derivatives, Phy a and Phy b, were detected in the tropical seaweeds. Seaweeds from the tropical area were dried before the analysis; therefore, most of the Chl a and Chl b in the tropical seaweed samples would have decomposed to produce corresponding derivatives, Phy a and Phy b, respectively. The heating treatments on seaweeds cause the central magnesium ion losses in the chlorophylls, which convert into degradation products, i.e., pheophytins [21,66]. Therefore, considering the degradation of Chl a and Chl b during the treatment of the tropical seaweeds, the major chlorophylls may be considered to be Chl a and Chl c for Ochrophyta, Chl a and Chl b for Chlorophyta, and Chl a for Rhodophyta. This study is in accordance with another study by Chen et al. [43].

In the present study, Chl c was found in two species of Rhodophyta, *C. crassicaulis* and *M. japonica* (Table 2). This finding is noteworthy because Chl c has been regarded as a specific chlorophyll in Ochrophyta [43,67]. Wilhelm [68] detected Chl c in Chlorophyta, namely, *Mantoniella comigrates*; however, there has been no report on the presence of Chl c in Rhodophyta. Chl c is also found in diatoms as their major chlorophyll. Diatoms use the blue-green spectral region for photosynthetic energy. They have Chl a and Chl c as their major chlorophylls and Fx as their major carotenoid. In the diatoms, the fucoxanthin–chlorophyll protein complex is mainly responsible for light-harvesting [69,70]. The present study suggests that the fucoxanthin–chlorophyll protein complex may be the key molecular complex for light harvesting, not only in diatoms, but also in several kinds of seaweeds [69,71].

As described above, the content of chlorophylls and their derivatives mainly varied among the seaweed phyla, but not with the different locations. The same trend was also found in the carotenoids composition (Table 3). The carotenoid content in each phylum is related to the difference in the carotenoid biosynthesis of each species [59]. Ochrophyta species were rich in Fx, with a considerable amount of β-Car and Zx and with a small amount of Vx. The presence of Fx, Zx, and Vx in Ochrophyta suggests the involvement of the xanthophyll-cycle pathway in carotenoid biosynthesis in this phylum [14,59]. On the other hand, α-Car and Lut were detected as major carotenoids in Chlorophyta and Rhodophyta. The presence of both carotenoids is closely related to the presence of the α-Car pathway in Rhodophyta and Chlorophyta [72]. Two species (*C. yendoi* and *M. japonica*) of Rhodophyta analyzed in this study lacked Zx. The lack of Zx may be related to photo-acclimation in these species [73]. 

Chlorophylls and carotenoids have been considered as the most important biomolecules for photosynthesis. In addition, both pigments have attracted major interest from biochemists and nutritionists alike, because they are known to have significant biochemical and physiological effects, and primarily exhibit a positive influence on human nutrition and health [28]. Specifically, much attention has been paid to the antioxidant activity of chlorophylls and carotenoids. Carotenoids are one of the most famous natural antioxidants, together with tocopherols and polyphenols. They are regarded as the most efficient natural quenchers of singlet oxygen (^1^O_2_), and this effect has been attributed to a physical mechanism where the excess energy of singlet oxygen is transferred to conjugated double bonds of carotenoids [19]. In addition to the singlet oxygen quenching ability, carotenoids can scavenge free radicals. The reactions with free radicals are much more complex than those with singlet oxygen. Chlorophylls are also known to scavenge free radicals, and the effect may be strongly related to their chemical structure, especially the porphyrin ring, phytyl chain, and extended system of conjugated double bonds [28]. 

The strong ability of these seaweed pigments to quench singlet oxygen and/or to scavenge free radicals has been suggested as the main mechanism by which they afford their health benefits [19,22,28,74]. The seaweed lipids examined in the present study contained carotenoids and chlorophylls as major lipid soluble antioxidants. In addition, α-Toc, the most popular natural antioxidant, was detected in these lipids. Therefore, the seaweed lipids may be a potential source of natural antioxidants that can be used as cosmetic and food ingredients [70,75]. α-Toc is a major tocopherol analogue found in seaweeds [45]. In this study, the α-Toc content in two species of seaweeds from the Sargassaceae family (*S. fusiforme* and *S. horneri*) was higher than that of other seaweeds, except for *C. yendoi* (Table 6). This result is in accordance with previous studies [76,77]. The present study also suggests the relatively lower α-Toc level in Chlorophyta seaweeds (*C. lentillifera*, *U. reticulata*, *U. australis*, *U. intestinalis*) as compared with the other phyla, Ochrophyta and Rhodophyta.

Table 4 together with Tables S1a, S1b and S1c show the FAs composition of seaweed lipids. The composition was strongly affected by the sampling location, as seen in the TL analysis. As shown in Table 4, total PUFAs were the most dominant FAs class in the temperate seaweeds, and the level was higher than that in the tropical seaweeds. In the seaweeds collected from the tropical area, SFAs were a major FAs class. This result is consistent with other studies [14,48,49,78]. Gerasimenko and Logvinov [79] reported that FAs content and composition in seaweeds differed not only by species, but also by seawater temperature. The lower temperature of seaweed habitats results in the high accumulation of *n-3* PUFAs for accelerating cell metabolism, especially during the winter [38]. The present study revealed that three phyla grown in the temperate area greatly accumulated EPA and SDA in Ochrophyta, ALA and SDA in Chlorophyta, and EPA in Rhodophyta, while these *n-3* PUFAs levels were relatively lower in the corresponding phyla grown in the tropical area (Tables S1a, S1b and S1c). On the other hand, there was not much difference in the *n-6* PUFAs levels between the seaweeds collected from the temperate and the tropical areas. The higher level of total PUFAs in temperate seaweeds may be derived from the higher accumulation rate of *n-3* PUFAs. 

It is known that TL content and fatty acids composition varies seasonally. For example, the seasonal changes of fatty acids composition have been determined in *Egregia menziesii* (Turner) [80], *S. japonica* [81,82], *C. costata* (Turner) [83], *Stephanocystis hakodatensis* [38], *S. horneri* [38], *S. oligocystum* [78], and *U. pinnatifida* (Harvey) [84]. In all cases, the higher level of total n-3PUFAs has been found during winter or spring; this is the growing period for these seaweeds. Seasonal variation was also found in photosynthetic pigments, such as chlorophylls and carotenoids [38,85,86,87,88,89,90]. In the present study, no concern has been given to the seasonal changes in the seaweed pigments. These pigments are strongly related to the growing rate of each seaweed species. Therefore, much attention has been paid to the relationship between the growth rate of seaweeds and the level of lipid compounds, especially focusing on the photosynthetic pigments such as chlorophylls and carotenoids.

The lower *n-6*/*n-3* PUFAs ratio in dietary lipids has been considered to reduce the risk for many kinds of chronic diseases [91]. Although, a balanced ratio between *n-6* and *n-3* PUFAs, usually 1:2 to 1:4 (*w*/*w*) [30], has been recommended. However, in modern society, intake of the *n-6* PUFAs is much lower than that of the *n-3* PUFAs, resulting in the increase in the *n-6*/*n-3* PUFAs ratio. Most of the seaweeds, except for *G. longissima*, showed a lower *n-6*/*n-3* ratio, mostly less than 1.0 (Table 5). In addition, TI and AI values in all seaweeds were less than 3, especially in the temperate seaweeds. The lower TI and AI values of temperate seaweeds are because of the considerable accumulation of *n-3* PUFAs in the temperate seaweeds. TI and AI values found in the present study were lower than those in Indian *macrolagae*, namely, *Gracillaria salicornia*, *Sarconema cinaioides*, *Hypnea spinella*, and *Laurencia dendroidea* [49]. Overall, all indices shown in Table 5 indicate that seaweed lipids analyzed in the present study, especially those from the temperate seaweeds, have a cardio-protective FA composition. Prabhasankar et al. [92] reported the change in the *n-6*/*n-3* ratio of pasta lipids after incorporation of the powder of brown seaweed collected from the temperate area. When 10% of the brown seaweed powder was incorporated in the pasta, the *n-6*/*n-3* ratio changed from 15.2 to 3.4. This result indicates that a drastic change could be found in the low-fat food by mixing seaweed powder rich in *n-3* PUFAs.

## 4. Materials and Methods 

### 4.1. Materials

Standards for Chl a, Phy a, Phy b, and β-Car were obtained from Wako Pure Chemicals (Tokyo, Japan). Chl b and Chl c1 were purchased from Sigma-Aldrich Japan Co. (Tokyo, Japan) and DHI Laboratory Products (Hørsholm, Denmark), respectively. α-Carotene, Lut, Zx, Vx, and Nx were purchased from Carote *Nature* GmbH (Münsingen, Switzerland). dl-α-Tocopherol (α-Toc) was obtained from Kanto Chemical Co. Inc. (Tokyo, Japan). Fx standard for use in this study was purified from the TL content of *S. horneri*, as described previously [20]. Tricosanoic acid (C23:0), used as an internal standard in the gas chromatography (GC) analysis, was obtained from Sigma-Aldrich (Tokyo, Japan). High-performance liquid chromatography (HPLC) grade solvents were used for the HPLC analysis and purchased from Wako Pure Chemicals, Ltd. (Osaka, Japan). All other solvents and chemicals used in the study were of analytical grade.

### 4.2. Sample Collection and Handling

Seaweeds were collected from two different regions, Indonesia (tropic) and Japan (temperate). The 15 seaweeds including harvesting locations, phylum, family, scientific and local name, and date of collection are listed in Table 1. After collection, tropical seaweeds were washed, air-dried at room temperature (3–5 days; 27–30 °C) to reduce their water content, and transported to the laboratory. Temperate seaweeds were kept in plastic bags, stored on ice, and transported to the laboratory. These samples were then stored in a freezer (−30 °C). 

### 4.3. Moisture Determination and TL Extraction

Before moisture determination, all temperate seaweed samples were thawed and washed several times with running tap water until there were no debris, sand, or other contaminants. Before extraction, the moisture content of 15 seaweeds species was estimated with an oven-drying method, as described by Gómez-Ordóñez et al. [93], and used for expressing TL, chlorophylls, carotenoids, *n-3* PUFAs, and α-Toc content in seaweed samples on a DW basis. The TL content from the seaweeds was obtained by overnight extraction with a ten-fold portion of ethanol (*w*/*v*). For the extraction, the samples were crudely cut into small pieces. In the case of tropical seaweeds, a nine-fold portion of water was added to the washed sample before the ethanol extraction. After immersion for one hour, the sample was subjected to the ethanol extraction, while the temperate raw seaweed sample was directly subjected to the ethanol extraction. The filtrate obtained by the extraction was filtered with Advantec No. 2 filter paper (Advantec Toyo Kaisha Ltd., Tokyo, Japan), and the residue was subjected to a further overnight extraction with the same solvent proportion. Both filtrates were combined and were vacuum-concentrated at 30 °C using a rotary evaporator to obtain the ethanol extract (dark green mass). The ethanol extract was dissolved in a chloroform/methanol/water solution with a ratio of 10:5:3 (*v*/*v*/*v*), and the solution was placed into a separatory funnel. After allowing the funnel to stand overnight, the solution was separated into two layers. The lower layer evaporated under reduced pressure in a rotary evaporator. The last traces of the solvent and water were removed using nitrogen and using a high-powered vacuum to obtain the seaweed TL content. The TL content was dissolved in ethanol and stored at −30 °C for further analysis. 

### 4.4. Pigment Analysis

The contents of chlorophylls, their derivatives, and carotenoids in the seaweed TL content were analyzed using HPLC. HPLC was performed with a Hitachi HPLC La Chrome system (Hitachi Seisakusho Co., Tokyo, Japan) equipped with an auto sampler (L-2200), pump (L-2130), column oven (L-2300), and photodiode array detector (L-2455). An aliquot of the TL content was weighed and dissolved in acetone. The sample solution was filtered with a 0.45 μm membrane filter of polytetrafluoroethylene (PTFE) (Ekicrodisc 13CR; Nippon Genetics Co. Ltd., Tokyo, Japan) and subjected to HPLC analysis. The analysis was performed on an octadecylsilyl (ODS) column (TSK-gel ODS 80-Ts, 250 × 4.6 mm i.d., 5 μm particle size; Tosoh, Japan) protected with a guard column (15 × 3.2 mm) with the same stationary phase. The mobile phase consisted of methanol/acetonitrile/1 M ammonium acetate (5:3:2, *v*/*v*/*v*) (A) and acetonitrile/ethyl acetate (1:1, *v*/*v*) (B) [94] with slight modification. A gradient elution procedure was programmed as follows: 0–2 min, 100:0 (A/B, *v*/*v*); 2–28 min, a linearly elution gradient from A to B; and 28–37 min, 0:100 (A/B, *v*/*v*). The flow rate was kept at 1.0 mL min^−1^. The UV/visible spectra absorption was recorded from 350 to 800 nm with a photodiode array detector, and sequential detection was carried out at 410, 430, 450, and 666 nm [43]. Quantification of chlorophylls, their derivatives, and carotenoids was performed with the corresponding calibration curves. A calibration curve for each pigment was prepared using an authentic standard.

### 4.5. Fatty Acids Analysis

The fatty acids composition of the TL content was determined by GC after conversion of fatty acyl groups in the lipid to their methyl esters. The fatty acid methyl esters (FAMEs) were prepared as per the method by Prevot and Mordret [95], with a slight modification. Briefly, 1 mL *n*-hexane containing an internal standard (tricosanoic acid, C23:0) and 0.2 mL 2 M NaOH in methanol were added to an aliquot of the TL content (*ca.* 10 mg), vortexed for 10 seconds, and incubated at 50 °C for 30 seconds. After the incubation, 0.2 mL 2 M HCl in methanol solution was added to the solution and vortexed for 60 seconds. The mixture was separated by centrifugation at 1000× *g* for five minutes. The upper hexane layer containing FAMEs was recovered and subjected to GC. The FAMEs analysis was performed on a Shimadzu GC-2014 (Shimadzu Seisakusho, Kyoto, Japan) equipped with a flame ionization detector and a capillary column (Omegawax-320; 30 m × 0.32 mm i.d.; Supelco, Bellefonte, PA, USA). The detector, injector, and column temperatures were 260, 250, and 200 °C, respectively. The carrier gas was helium at a flow rate of 1.0 mL s^−1^. FA content was expressed as the relative weight percentage of the total FAs weight. Each *n-3* PUFAs level (mg∙g^−1^ TL content) was quantified by comparing the peak ratio to that of the internal standard (23:0). Total *n-3* PUFAs (mg∙g^−1^ DW) were calculated from the content of each *n-3* PUFAs in the TL content and the TL content g^−1^ DW. FAME peaks were identified by comparison of their retention time and log RRT with GLC-Reference standards by GC FID (Shimadzu GC-2014) after analysis. The fatty acids standard was prepared with GLC-Reference Standard, fatty acid methyl esters: GLC-462, and Echium oil, according to Susanto et al [34]. 

Several factors predicting the nutritional quality of the seaweed TL were calculated based on the formula as below [79,96,97]:

(1) $$n-6/n-3\;\mathrm{ratio}\;=\;\frac{\sum n-6\mathrm{PUFAs}}{\sum n-3\mathrm{PUFAs}}$$

(2) $$\mathrm{Atherogenicity}\;\mathrm{index}\;(\mathrm{AI})\;=\;\frac{C12:0+4\times C14:0+C16:0}{\sum \mathrm{MUFAs}+\sum n-6\;\mathrm{PUFAs}+\sum n-3\;\mathrm{PUFAs}}$$

(3) Thrombogenicity index (TI) = C14:0+C16:0+C18:0(0.5×∑MUFAs)+(0.5×∑n-6 PUFAs)+(3×∑n-3 PUFAs)+∑n-3 PUFAs∑n-6 PUFAs

(4) $$\mathrm{Fatty}\;\mathrm{acids}\;\mathrm{hypocholesterolemic}/\mathrm{hypercholesterolemic}\;\mathrm{ratio}\;(h/H)\;=\;\frac{C18:1n-9+C18:2n-6+C20:4n-6+C18:3n-3+C20:5n-3}{C14:0+C16:0}$$

(5) Unsaturation Index (UI) =∑(% PUFAs×DB); DB is the number of double bonds.

### 4.6. α-Toc Analysis

The content of α-Toc in the TL content was analyzed by HPLC with a silica column (Mightysil Si 60 silica, 250 × 4.6 mm i.d.; Kanto Chemical Co., Ltd., Tokyo, Japan). HPLC was carried out with the same system as described in the pigment analysis, except that the fluorescence detector (Hitachi L-7485) was used for the peak detection. The mobile phase was hexane/2-propanol (99.2:0.8, *v*/*v*) at a flow rate of 1.0 mL min^−1^. The fluorescence detector was set at Ex. 298 nm and Em. 325 nm.

### 4.7. Data Analysis 

All analytical data were conducted in triplicate and the mean values are presented. The PCA was performed using JMP 14.0 statistical analysis. Two principal components based on the fatty acids composition and group (SFAs, MUFAs, PUFAs, *n*-3 PUFAs, and *n*-6 PUFAs) were used to make clear the relationship between different sampling areas and seaweed phyla. Fatty acids composition (supplementary data) and FAs group composition (Table 4) were used for the multivariate analysis. However, an insignificant amount of fatty acids was not included in this analysis, these fatty acids were dodecanoic acid (C12:0), tridecanoic acid (C13:0), 7-tetradecenoic acid (C14:1*n*-7), pentadecanoic acid (C15:0), 8-pentadecenoic acid (C15:1*n*-7), heptadecanoic acid (C17:0), 10-heptadecenoic acid (C17:1*n*-7), 11,14,17-eicosatrienoic acid (C20:3*n-3*), docosanoic acid (C22:0), 13-docosaenoic acid (C22:1*n*-9), 13,16-docosadieonic acid (C22:2*n-6*), tetradocosanoic acid (C24:0), and 17-tetracosaenoic acid (C24:1*n*-7). 

## 5. Conclusions

In conclusion, the present study clearly shows the major factors affecting the variation of seaweed lipid compounds. Liphophilic pigments were explained by seaweeds’ phyla, while the geographical location influenced fatty acids composition. Lipid soluble pigments were one of the major compounds of interest in the present study. Among these pigments, relatively little attention has been paid to chlorophylls, despite the fact that chlorophylls are abundant in our diet and their biological significance has been recognized for a long time. The results for the compositions of chlorophylls and their derivatives will provide valuable information for the application of each seaweed’s lipids as a good source of these chlorophyll compounds and other lipid soluble compounds, such as carotenoids and PUFAs.

## Figures and Tables

**Table 1:** List of seaweeds examined and total lipids (TL) content of these seaweeds *.

Harvesting Location	Phylum	Family	Seaweeds	Local Name	Collection Date	Total Lipids (mg∙g^−1^ DW)
Tual, Indonesia ^a,d^	Chlorophyta	Caulerpaceae	*Caulerpa lentillifera*	*Lat*	Feb-17	15.75 ± 0.82
Hakodate, Japan ^b,f^	Chlorophyta	Ulvaceae	*Ulva australis*	*Anaaosa*	Jun-17	62.48 ± 3.05
Hakodate, Japan ^b,f^	Chlorophyta	Ulvaceae	*Ulva intestinalis*	*Bouaonori*	Jun-17	37.46 ± 7.56
Tual, Indonesia ^a,d^	Chlorophyta	Ulvaceae	*Ulva reticulata*	*Lumut daun*	May-17	22.70 ± 3.01
Hakodate, Japan ^b,f^	Ochrophyta	Agaraceae	*Costaria costata*	*Sujime*	May-17	33.71 ± 1.86
Hakodate, Japan ^b,f^	Ochrophyta	Alariaceae	*Undaria pinnatifida*	*Wakame*	May-17	58.10 ± 4.56
Hakodate, Japan ^b,f^	Ochrophyta	Laminariaceae	*Saccharina japonica*	*Konbu*	May-17	37.42 ± 6.23
Tual, Indonesia ^a,c,d^	Ochrophyta	Sargassaceae	*Sargassum aquifolium* ^a^	*Pama*	Feb-17	20.87 ± 0.70
Hakodate, Japan ^b,f^	Ochrophyta	Sargassaceae	*Sargassum fusiforme*	*Hijiki*	May-17	48.54 ± 1.61
Iwate, Japan ^b,g^	Ochrophyta	Sargassaceae	*Sargassum horneri*	*Akamoku*	May-15	55.97 ± 2.51
Hakodate, Japan ^b,f^	Rhodophyta	Endocladiaceae	*Gloiopeltis furcat* *a*	*Fukurofunori*	May-17	8.94 ± 1.45
Hakodate, Japan ^b,f^	Rhodophyta	Gigartinaceae	*Chondrus yendoi*	*Kurohaginnansou*	Jun-17	6.91 ± 0.21
Hakodate, Japan ^b,f^	Rhodophyta	Gigartinaceae	*Mazzaella japonica*	*Akabaginnansou*	Jun-17	14.25 ± 0.30
Jepara, Indonesia ^a,e^	Rhodophyta	Gracilariaceae	*Gracilariopsis longissima*	*agar-agar*	May-17	8.86 ± 0.15
Hakodate, Japan ^b,f^	Rhodophyta	Rhodomelaceae	*Chondria crassicaulis*	*Yuna*	Jun-17	49.77 ± 2.40

* The data value is expressed as the mean ± SD of three replicate measurements; ^a^ fresh samples collected, immediately washed, air dried, and kept in refrigerator (tropical seaweeds); ^b^ fresh samples collected, immediately kept in refrigerator (temperate seaweeds); ^c^ non-edible seaweeds; ^d^ latitudes/longitudes: 5° 37′27.3396″ S 132°43′22.3392″ E; ^e^ latitudes/longitudes: 6.5868° S, 110.6444° E; ^f^ latitudes/longitudes: 41°45′ N, 140°49′ E; ^g^ latitudes/longitudes: 39°28′46.5″ N 142°00′11.9″ E. DW, dry weight.

**Table 2:** Composition of chlorophylls and pheophytins in 15 species of seaweeds (mg∙100 g^−1^ DW) *.

Seaweeds	Chl a	Phy a	Chl b	Phy b	Chl c (c1 + c2) ^b^	Total Chlorophylls
*Caulerpa lentillifera*	n.d.	118.68 ± 44.41	16.83 ± 4.60	90.28 ± 8.97	n.d.	225.79
*Ulva australis*	81.90 ± 4.94	26.17 ± 3.27	64.45 ± 9.36	n.d.	n.d.	172.52
*Ulva intestinalis*	115.57 ± 51.28	165.98 ± 14.70	101.50 ± 29.21	64.38 ± 11.30	n.d.	447.43
*Ulva reticulata*	n.d.	115.85 ± 21.17	18.04 ± 0.61	267.69 ± 158.86	n.d.	401.58
*Costaria costata*	16.28 ± 3.80	291.92 ± 67.84	n.d.	n.d.	21.78 ± 0.84	329.97
*Undaria pinnatifida*	54.67 ± 14.60	423.42 ± 57.72	n.d.	n.d.	38.58 ± 5.03	518.67
*Saccharina japonica*	26.13 ± 16.79	425.42 ± 19.17	n.d.	n.d.	17.11 ± 2.91	469.24
*Sargassum aquifolium* ^a^	12.17 ± 4.02	149.64 ± 56.00	n.d.	n.d.	2.12 ± 0.21	163.93
*Sargassum fusiforme*	210.72 ± 43.63	107.59 ± 30.30	n.d.	n.d.	18.20 ± 0.34	336.51
*Sargassum horneri*	268.82 ± 59.93	220.86 ± 22.84	n.d.	n.d.	23.29 ± 4.9	512.97
*Gloiopeltis furcata*	2.98 ± 1.96	28.21 ± 4.92	n.d.	n.d.	n.d.	31.19
*Chondrus yendoi*	4.77 ± 2.10	29.29 ± 2.21	n.d.	n.d.	n.d.	34.06
*Mazzaella japonica*	6.70 ± 1.28	16.25 ± 1.85	n.d.	n.d.	7.63 ± 1.25	30.58
*Gracilariopsis longissima*	n.d.	14.66 ± 2.24	n.d.	n.d.	n.d.	14.66
*Chondria crassicaulis*	1.74 ± 1.19	322.72 ± 56.48	n.d.	n.d.	10.18 ± 1.29	334.64

* The data value is expressed as the mean ± SD of three replicate measurements; ^a^ non-edible seaweed; ^b^ Chl c composes of Chl c1 and Chl c2. However, both chlorophylls could not be separated on HPLC to be detected as one peak; therefore, Chl c1 and Chl c2 were expressed as Chl c altogether in the present study. Quantification of Chl c was done using the calibration curve of c1 standard, because Chl c1 has been reported to be the major chlorophyll c in brown seaweeds [37]. n.d. = not detected. Abbreviations: Chl a: chlorophyll a; Chl b: chlorophyll b; Chl c: chlorophyll c; Phy a: pheophytin a; Phy b: pheophytin b.

**Table 3:** Carotenoids content of 15 species of seaweeds (mg∙100g^−1^ DW) *.

Seaweeds	β-Car	α-Car	Zx	Lut	Vx	Nx	Fx	Total Carotenoids
*Caulerpa lentillifera*	1.84 ± 0.70	17.15 ± 2.96	n.d.	1.02 ± 0.53	n.d.	n.d.	n.d.	20.01
*Ulva australis*	58.24 ± 6.52	63.01 ± 0.04	n.d.	124.82 ± 13.14	23.30 ± 0.74	185.56 ± 59.65	n.d.	454.93
*Ulva intestinalis*	39.91 ± 9.54	17.83 ± 6.66	n.d.	77.78 ± 18.14	13.15 ± 4.19	153.70 ± 84.20	n.d.	302.37
*Ulva reticulata*	3.89 ± 0.39	4.83 ± 0.35	n.d.	14.12 ± 2.85	n.d.	30.63 ± 7.84	n.d.	53.47
*Costaria costata*	7.31 ± 0.86	n.d.	n.d.	n.d.	1.45 ± 0.19	n.d.	97.60 ± 12.87	106.36
*Undaria pinnatifida*	25.58 ± 3.71	n.d.	12.03 ± 3.99	n.d.	3.51 ± 0.61	n.d.	169.48 ± 12.98	210.61
*Saccharina japonica*	18.10 ± 0.61	n.d.	3.04 ± 0.13	n.d.	1.47 ± 0.25	n.d.	154.71 ± 11.29	177.32
*Sargassum aquifolium* ^a^	12.51 ± 1.24	n.d.	2.45 ± 0.32	n.d.	n.d.	n.d.	108.44 ± 9.17	123.40
*Sargassum fusiforme*	44.70 ± 7.52	n.d.	7.06 ± 1.65	n.d.	12.15 ± 2.45	n.d.	140.93 ± 12.98	204.84
*Sargassum horneri*	42.70 ± 6.70	n.d.	29.21 ± 2.72	n.d.	4.74 ± 0.98	n.d.	216.50 ± 31.97	293.15
*Gloiopeltis furcata*	8.99 ± 2.53	3.73 ± 0.70	0.47 ± 0.30	+(8.76 ± 2.31) ^c^	n.d.	n.d.	3.43 ± 0.48	25.38
*Chondrus yendoi*	2.07 ± 0.19	4.60 ± 0.47	n.d.	1.78 ± 0.81	n.d.	n.d.	2.57 ± 0.07	11.02
*Mazzaella japonica*	2.23 ± 0.18	1.95 ± 0.27	n.d.	0.62 ± 0.12	n.d.	n.d.	7.26 ± 0.42	12.06
*Gracilariopsis longissima*	1.12 ± 0.25	n.d.	+(0.75 ± 0.16) ^b^	n.d.	n.d.	n.d.	n.d.	1.87
*Chondria crassicaulis*	13.78 ± 2.40	8.19 ± 0.82	+(4.94 ± 1.66) ^b^	n.d.	n.d.	n.d.	67.76 ± 9.63	94.67

*The data value is expressed as the mean ± SD of three replicate measurements; ^a^ non-edible seaweeds; ^b^ Zx and Lut were calculated from the calibration curve of Zx standard; ^c^ Lut and Zx were calculated from the calibration curve of Lut standard. n.d. = not detected. Abbreviations: β-Car: β-carotene; α-Car: α-carotene; Zx: zeaxanthin; Lut: lutein; Vx: violaxanthin; Nx: neoxanthin; Fx: fucoxanthin.

**Table 4:** Fatty acids composition (weight % of total fatty acids (FAs)) *.

Seaweeds	Σ C16	Σ C18	Σ C20	Σ SFAs	Σ MUFAs	Σ PUFAs	Σ *n-3* PUFAs	Σ *n-6* PUFAs	Σ *n-3* PUFAs ^b^
*Caulerpa lentillifera*	33.69 ± 0.64	13.57 ± 0.91	2.84 ± 0.53	29.80 ± 1.65	9.08 ± 2.75	13.06 ± 0.32	7.31 ± 0.41	5.75 ± 0.10	0.01
*Ulva australis*	24.37 ± 0.35	35.41 ± 0.44	4.23 ± 0.08	25.67 ± 0.47	3.45 ± 0.24	36.23 ± 0.82	29.00 ± 0.66	7.22 ± 0.22	6.83
*Ulva intestinalis*	23.26 ± 1.27	36.61 ± 0.87	7.72 ± 2.31	24.37 ± 1.21	4.24 ± 0.87	39.16 ± 1.54	29.03 ± 2.02	10.14 ± 0.65	9.84
*Ulva reticulata*	44.66 ± 0.38	23.96 ± 0.38	6.01 ± 0.63	43.01 ± 0.46	6.88 ± 0.42	25.75 ± 0.52	23.25 ± 0.36	2.50 ± 0.43	0.02
*Costaria costata*	23.88 ± 1.12	30.84 ± 1.13	22.00 ± 1.92	37.46 ± 2.02	15.92 ± 1.46	36.59 ± 2.62	15.38 ± 2.15	21.21 ± 0.79	0.53
*Undaria pinnatifida*	25.73 ± 1.22	38.31 ± 4.44	23.01 ± 0.39	31.12 ± 1.75	14.07 ± 0.90	48.19 ± 5.72	28.94 ± 5.71	19.25 ± 0.30	4.66
*Saccharina japonica*	16.79 ± 0.75	38.83 ± 0.79	26.93 ± 0.18	24.35 ± 1.18	15.76 ± 0.64	51.28 ± 0.61	31.24 ± 1.91	20.04 ± 1.57	2.26
*Sargassum aquifolium* ^a^	48.45 ± 2.70	21.83 ± 0.75	13.46 ± 0.30	46.80 ± 5.31	18.88 ± 1.40	22.90 ± 0.89	6.26 ± 0.37	16.64 ± 0.52	0.10
*Sargassum fusiforme*	24.80 ± 0.52	27.01 ± 0.43	30.38 ± 0.45	27.62 ± 0.58	11.95 ± 0.22	47.32 ± 1.08	30.22 ± 0.78	17.11 ± 0.27	2.66
*Sargassum horneri*	26.35 ± 1.30	30.25 ± 0.96	30.49 ± 1.14	26.98 ± 1.49	14.24 ± 0.25	49.00 ± 2.36	26.97 ± 1.64	22.03 ± 1.16	4.03
*Gloiopeltis furcata*	23.34 ± 1.31	19.36 ± 0.11	44.89 ± 2.24	26.97 ± 1.45	18.05 ± 0.23	45.33 ± 2.36	38.11 ± 2.12	7.22 ± 0.24	0.46
*Chondrus yendoi*	33.12 ± 0.48	12.60 ± 0.23	42.35 ± 0.89	37.28 ± 0.62	9.89 ± 0.19	43.91 ± 0.85	24.23 ± 0.81	19.68 ± 0.21	0.68
*Mazzaella japonica*	40.50 ± 0.38	16.42 ± 0.05	30.12 ± 0.29	45.29 ± 0.51	14.49 ± 0.61	31.21 ± 0.72	20.05 ± 0.50	11.15 ± 0.24	0.10
*Gracilariopsis longissima*	46.44 ± 0.91	10.16 ± 0.50	15.21 ± 0.69	47.78 ± 2.36	11.18 ± 1.73	13.90 ± 0.64	1.63 ± 0.26	12.28 ± 0.71	0.01
*Chondria crassicaulis*	38.41 ± 1.54	19.93 ± 1.20	18.50 ± 0.69	36.88 ± 1.23	20.49 ± 0.88	28.15 ± 1.37	19.82 ± 0.71	8.33 ± 0.82	2.73

* The data value is expressed as the mean ± SD of three replicate measurements; ^a^ non-edible seaweeds; ^b^ absolute content of total *n*-3 PUFAs (mg∙g^−1^ DW). Abbreviations: SFAs: saturated fatty acids; MUFAs: mono unsaturated fatty acids; PUFAs: polyunsaturated fatty acids.

**Table 5:** Nutritional quality index of different seaweeds judged from fatty acids composition.

Seaweeds	*n-6*/*n-3* PUFAs	AI	TI	h/H	UI
*Caulerpa lentillifera*	0.79 ± 0.05	1.53 ± 0.28	0.96 ± 0.13	0.44 ± 0.04	3.00 ± 0.07
*Ulva australis*	0.25 ± 0.01	0.64 ± 0.01	0.26 ± 0.01	1.04 ± 0.02	3.27 ± 0.01
*Ulva intestinalis*	0.35 ± 0.05	0.57 ± 0.04	0.23 ± 0.01	1.39 ± 0.13	3.19 ± 0.03
*Ulva reticulata*	0.11 ± 0.02	1.31 ± 0.05	0.35 ± 0.27	0.33 ± 0.02	3.67 ± 0.01
*Costaria costata*	1.40 ± 0.18	1.37 ± 0.16	0.54 ± 0.08	1.28 ± 0.12	3.63 ± 0.06
*Undaria pinnatifida*	0.68 ± 0.13	0.69 ± 0.08	0.28 ± 0.06	1.66 ± 0.23	3.71 ± 0.04
*Saccharina japonica*	0.65 ± 0.09	0.67 ± 0.04	0.20 ± 0.02	2.23 ± 0.08	3.88 ± 0.04
*Sargassum aquifolium* ^a^	2.66 ± 0.07	1.42 ± 0.25	1.23 ± 0.20	0.67 ± 0.07	3.40 ± 0.01
*Sargassum fusiforme*	0.57 ± 0.01	0.62 ± 0.02	0.24 ± 0.01	1.76 ± 0.06	3.93 ± 0.00
*Sargassum horneri*	0.82 ± 0.05	0.53 ± 0.05	0.26 ± 0.03	1.82 ± 0.16	3.86 ± 0.02
*Gloiopeltis furcata*	0.19 ± 0.00	0.50 ± 0.05	0.19 ± 0.02	2.46 ± 0.26	4.75 ± 0.01
*Chondrus yendoi*	0.81 ± 0.03	0.79 ± 0.03	0.41 ± 0.02	1.48 ± 0.05	4.46 ± 0.01
*Mazzaella japonica*	0.56 ± 0.01	1.14 ± 0.03	0.58 ± 0.03	0.97 ± 0.03	4.49 ± 0.00
*Gracilariopsis longissima*	7.69 ± 1.58	2.03 ± 0.24	2.80 ± 0.25	0.44 ± 0.03	3.87 ± 0.02
*Chondria crassicaulis*	0.42 ± 0.03	1.19 ± 0.09	0.47 ± 0.03	0.86 ± 0.05	4.13 ± 0.03

^a^ Non-edible seaweeds. Abbreviations: AI: atherogenicity index; TI: thrombogenicity index; h/H: fatty acids hypocholesterolemic/hypercholesterolemic ratio; UI: unsaturation index.

**Table 6:** α-Tocopherols content (mg∙100g^−1^ DW) *.

Seaweeds	α-Toc
*Caulerpa lentillifera*	0.87 ± 0.21
*Ulva australis*	0.44 ± 0.01
*Ulva intestinalis*	0.83 ± 0.06
*Ulva reticulata*	1.13 ± 0.08
*Costaria costata*	1.37 ± 0.10
*Undaria pinnatifida*	1.09 ± 0.05
*Saccharina japonica*	1.82 ± 0.02
*Sargassum aquifolium* ^a^	2.40 ± 0.02
*Sargassum fusiforme*	3.56 ± 0.08
*Sargassum horneri*	3.65 ± 0.031
*Gloiopeltis furcata*	2.71 ± 0.34
*Chondrus yendoi*	9.34 ± 0.19
*Mazzaella japonica*	1.72 ± 0.009
*Gracilariopsis longissima*	2.58 ± 0.015
*Chondria crassicaulis*	0.54 ± 0.009

* The data value is expressed as the mean ± SD of three replicate measurements, ^a^ non-edible seaweeds.

## Supplementary Materials

The following are available online at https://www.mdpi.com/1660-3397/17/11/630/s1, Table S1a: Fatty acids composition (weight % of total FAs) of Ochrophyta; Table S1b: Fatty acids composition (weight % of total FAs) of Chlorophyta; Table S1c: Fatty acids composition (weight % of total FAs) of Rhodophyta.
