# Supplementary material for: Variation in Lipid Components from 15 Species of Tropical and Temperate Seaweeds
Source: Mar Drugs. 2019 Nov 6;17(11):630. doi: 10.3390/md17110630 (PMC6891767; doi:10.3390/md17110630)
Supplement: Supplementary file 1 [file marinedrugs-17-00630-s001.pdf]

marine drugs

MDPI

SUPPLEMENTARY

Table S1a. Fatty acids composition (% of total FA) of Phaeophyta*

Fatty acids	*Sargassum aquifolium*[a]	*Costaria costata*	*Saccharina japonica*	*Undaria pinnatifida*	*Sargassum fusiforme*	*Sargassum honeri*
12:0	0.03±0.01	0.08±0.06	0.01±0.01	0.02±0.01	0.01±0.00	0.01±0.00
13:0	0.01±0.01	0.07±0.01	n.d.	0.01±0.00	n.d.	n.d.
14:0	4.82±0.76	12.47±1.35	7.53±0.67	5.23±0.21	3.63±0.10	2.65±0.17
14:1	0.004±0.01	0.12±0.02	0.06±0.01	0.01±0.00	0.06±0.00	0.03±0.01
15:0	0.37±0.05	0.34±0.03	0.33±0.01	0.44±0.01	0.40±0.01	0.20±0.02
15:1	n.d.	0.004±0.01	n.d.	n.d.	n.d.	n.d.
16:0	39.78±4.47	21.88±1.03	14.79±0.75	22.00±1.36	22.18±0.48	22.77±1.19
16:1	8.67±1.77	2.00±0.19	2.00±0.12	3.73±0.24	2.62±0.04	3.59±0.11
17:0	0.09±0.01	0.08±0.02	0.10±0.01	0.12±0.01	0.06±0.01	0.03±0.00
17:1n-7	0.18±0.08	0.11±0.01	0.81±0.04	0.50±0.05	0.56±0.02	0.20±0.03
18:0	0.66±0.08	0.70±0.13	0.50±0.01	1.62±0.14	0.32±0.03	0.34±0.04
18:1n-9	9.91±0.45	13.24±1.32	12.70±0.60	9.43±1.10	6.32±0.10	7.97±0.14
18:2n-6	5.05±0.24	8.74±0.39	6.26±0.30	7.61±0.10	3.07±0.05	6.21±0.37
18:3n-6	0.98±0.30	1.30±0.10	1.28±0.10	0.90±0.01	0.22±0.01	0.76±0.05
18:3n-3	3.13±0.19	2.63±0.32	5.27±0.25	7.43±4.23	9.38±0.19	5.07±0.08
18:4n-3	2.11±0.20	4.23±0.51	12.84±0.44	11.33±1.23	7.71±0.26	9.91±0.72
20:0	0.20±0.10	0.53±0.04	0.40±0.02	0.81±0.07	0.18±0.00	0.17±0.06
20:1n-9	n.d.	n.d.	0.01±0.01	0.01±0.00	2.38±0.09	2.38±0.14
20:2n-6	0.22±0.01	0.12±0.01	0.06±0.04	0.06±0.00	0.12±0.01	0.19±0.02
21:0	1.02±0.04	1.31±0.07	0.69±0.07	0.78±0.06	0.49±0.00	0.51±0.04
20:4n-6	10.40±0.23	10.96±0.51	12.44±1.23	10.55±0.37	13.68±0.21	14.87±0.73
20:3n-3	0.07±0.01	0.07±0.01	0.08±0.01	0.09±0.01	0.15±0.01	0.10±0.01
20:5n-3	0.96±0.06	8.36±1.37	13.04±1.34	10.08±0.58	12.96±0.35	11.89±0.92

22:0	0.30±0.05	0.01±0.01	n.d.	0.03±0.00	0.27±0.02	0.21±0.06
22:1n-9	n.d.	n.d.	0.004±0.01	0.02±0.01	0.02±0.01	0.01±0.01
22:2n-6	n.d.	0.10±0.02	0.01±0.01	0.12±0.18	0.01±0.01	0.01±0.00
22:5n-3	n.d.	0.10±0.03	n.d.	0.02±0.00	0.01±0.01	n.d.
22:6n-3	n.d.	n.d.	n.d.	0.004±0.01	n.d.	n.d.
23:0	n.d.	n.d.	n.d.	n.d.	n.d.	n.d.
24:0	0.18±0.03	0.01±0.01	0.01±0.01	0.07±0.00	0.10±0.01	0.09±0.03
24:1	0.12±0.02	0.45±0.01	0.18±0.08	0.37±0.04	n.d.	0.06±0.04
Identified FAs	89.12	89.97	91.39	93.38	86.89	90.22
Unidentified FAs	7.36	10.03	8.61	6.62	13.11	9.78

*The data value is expressed as the mean±SD of three replicate measurements; [a]non-edible seaweeds

Table S1b. Fatty acids composition (% of fatty acids) of Chlorophyta*

Fatty acids	*Caulerpa lentilifera*	*Ulva reticulata*	*Ulva australis*	*Ulva intestinalis*
12:0	0.06±0.02	Trace	0.08±0.06	n.d.
13:0	0.81±0.05	n.d.	0.02±0.01	0.003±0.01
14:0	1.68±0.36	0.34±0.29	0.54±0.01	0.93±0.15
14:1	n.d.	n.d.	n.d.	n.d.
15:0	0.21±0.04	0.21±0.18	0.08±0.01	0.12±0.02
15:1	n.d.	n.d.	n.d.	n.d.
16:0	25.70±6.02	27.51±23.48	23.19±0.19	20.90±1.47
16:1	10.43±3.71	2.46±2.10	1.18±0.16	2.36±1.20
17:0	0.19±0.17	0.15±0.13	0.17±0.01	0.21±0.08
17:1n-7	n.d.	0.25±0.21	0.23±0.02	0.10±0.02
18:0	1.14±0.22	0.28±0.24	0.83±0.44	0.51±0.55
18:1n-9	0.94±0.19	1.48±1.27	1.54±0.06	1.41±0.11
18:2n-6	2.60±0.58	1.15±0.98	5.98±0.18	7.59±1.19
18:3n-6	1.04±0.46	0.21±0.18	0.70±0.03	1.86±1.75
18:3n-3	4.67±0.93	5.63±4.80	16.15±0.33	18.44±2.46
18:4n-3	1.43±0.80	7.17±6.11	10.21±0.28	6.80±1.15
20:0	n.d.	0.15±0.14	0.02±0.01	0.56±0.43
20:1n-9	0.03±0.01	0.02±0.03	0.15±0.01	0.20±0.06
20:2n-6	0.14±0.03	n.d.	0.05±0.01	0.03±0.02
21:0	0.09±0.05	0.07±0.06	0.18±0.01	0.61±0.04
20:4n-6	0.99±0.26	0.38±0.47	0.49±0.02	0.66±0.10
20:3n-3	0.06±0.01	0.05±0.04	0.05±0.00	0.09±0.03
20:5n-3	0.37±0.10	0.561±0.48	0.60±0.03	2.20±0.47
22:0	0.07±0.01	0.50±0.42	0.35±0.02	0.33±0.06

22:1n-9	n.d.	n.d.	0.12±0.01	0.03±0.03
22:2n-6	n.d.	n.d.	n.d.	n.d.
22:5n-3	n.d.	2.03±1.73	1.98±0.05	1.44±0.17
22:6n-3	n.d.	n.d.	0.01±0.01	0.06±0.06
23:0	n.d.	n.d.	n.d.	1.17±2.03
24:0	0.39±0.21	0.03±0.06	0.01±0.01	0.20±0.08
24:1	0.03±0.03	0.26±0.23	0.23±0.00	0.14±0.12
Identified FAs	53.07	75.59	65.31	68.96
Unidentified FAs	31.34	16.13	34.67	31.64

*The data value is expressed as the mean±SD of three replicate measurements

8 **Tables S1c.** Fatty acids composition (% of fatty acids) of Rhodophyta*

Fatty acids	*Gracillariopsis longissimi*	*Chondria crassicaulis*	*Chondrus yendoi*	*Goliopeltis fusrcata*	*Mazaella japonica*
12:0	0.10±0.06	0.10±0.03	0.11±0.01	0.05±0.02	0.12±0.01
13:0	0.08±0.02	0.04±0.01	0.01±0.000	0	0.01±0.00
14:0	1.87±0.19	7.68±0.57	2.53±0.17	2.50±0.24	3.41±0.18
14:1	n.d.	0.03±0.03	n.d.	n.d.	n.d.
15:0	0.48±0.05	0.45±0.02	0.21±0.01	0.13±0.01	0.22±0.02
15:1	n.d.	n.d.	n.d.	n.d.	n.d.
16:0	43.03±0.83	26.75±1.04	32.40±0.49	21.40±1.25	38.31±0.27
16:1	3.41±0.87	11.66±0.71	0.72±0.02	1.95±0.07	2.19±0.19
17:0	0.19±0.07	0.09±0.01	0.15±0.02	0.07±0.03	0.19±0.02
17:1n-7	0.15±0.06	0.27±0.04	n.d	n.d	n.d
18:0	1.83±0.06	1.27±0.43	1.06±0.02	1.08±0.03	1.81±0.02
18:1n-9	7.10±0.48	8.04±0.16	8.96±0.15	15.42±0.11	11.45±0.08
18:2n-6	0.54±0.03	3.05±0.09	1.55±0.03	1.70±0.08	1.71±0.01
18:3n-6	0.12±0.02	1.72±0.85	0.80±0.02	0.40±0.01	0.62±0.01
18:3n-3	0.53±0.04	1.93±0.08	0.10±0.01	0.62±0.05	0.35±0.02
18:4n-3	0.05±0.08	3.92±0.13	0.12±0.01	0.15±0.02	0.48±0.02
20:0	0.10±0.01	0.14±0.01	0.004±0.01	0.01±0.00	0.09±0.01
20:1n-9	0.05±0.01	0.22±0.07	0.09±0.00	0.14±0.04	0.08±0.01
20:2n-6	0.20±0.02	0.22±0.05	0.07±0.00	0.18±0.01	0.16±0.00
21:0	0.66±0.08	0.27±0.02	0.79±0.03	1.54±0.07	0.89±0.00
20:4n-6	11.41±0.70	3.33±0.18	17.26±0.17	4.93±0.17	8.67±0.24
20:3n-3	0.52±0.22	0.04±0.03	0.05±0.00	0.02±0.00	0.01±0.01
20:5n-3	0.21±0.17	13.08±0.49	23.67±0.83	35.81±2.02	18.28±0.49

22:0	0.09±0.01	0.02±0.02	0.003±0.01	0.01±0.00	0.05±0.02
22:1n-9	0.03±0.03	0.04±0.03	0.06±0.06	0.32±0.01	0.15±0.01
22:2n-6	0.09±0.01	0.004±0.01	n.d.	n.d.	n.d.
22:5n-3	0.01±0.02	0.81±0.17	0.08±0.01	0.160±0.04	0.19±0.01
22:6n-3	0.32±0.08	0.05±0.04	0.20±0.01	1.36±0.07	0.74±0.04
23:0	n.d.	n.d.	n.d.	n.d.	n.d.
24:0	1.18±1.46	0.06±0.05	0.01±0.018	0.19±0.02	0.18±0.03
24:1	0.43±0.40	0.23±0.03	0.05±0.00	0.22±0.05	0.63±0.45
Identified FAs	74.26	85.51	91.68	90.34	90.98
Unidentified FAs	16.49	14.49	8.92	9.66	9.02

*The data value is expressed as the mean±SD of three replicate measurements
